# mid-DeepLabv3+: A Novel Approach for Image Semantic Segmentation Applied to African Food Dietary Assessments

**DOI:** 10.3390/s24010209

**Published:** 2023-12-29

**Authors:** Thierry Roland Baban A Erep, Lotfi Chaari

**Affiliations:** Toulouse INP, University of Toulouse, Institut de Recherche en Informatique de Toulouse, 31400 Toulouse, France

**Keywords:** food segmentation, semantic segmentation, CamerFood10 dataset, CNN

## Abstract

Recent decades have witnessed the development of vision-based dietary assessment (VBDA) systems. These systems generally consist of three main stages: food image analysis, portion estimation, and nutrient derivation. The effectiveness of the initial step is highly dependent on the use of accurate segmentation and image recognition models and the availability of high-quality training datasets. Food image segmentation still faces various challenges, and most existing research focuses mainly on Asian and Western food images. For this reason, this study is based on food images from sub-Saharan Africa, which pose their own problems, such as inter-class similarity and dishes with mixed-class food. This work focuses on the first stage of VBDAs, where we introduce two notable contributions. Firstly, we propose mid-DeepLabv3+, an enhanced food image segmentation model based on DeepLabv3+ with a ResNet50 backbone. Our approach involves adding a middle layer in the decoder path and SimAM after each extracted backbone feature layer. Secondly, we present CamerFood10, the first food image dataset specifically designed for sub-Saharan African food segmentation. It includes 10 classes of the most consumed food items in Cameroon. On our dataset, mid-DeepLabv3+ outperforms benchmark convolutional neural network models for semantic image segmentation, with an mIoU (mean Intersection over Union) of 65.20%, representing a +10.74% improvement over DeepLabv3+ with the same backbone.

## 1. Introduction

Despite significant advancements in the medical field, the prevalence of Non-Communicable Diseases (NCDs), such as cardiovascular diseases, cancers, chronic respiratory diseases, obesity, and diabetes, remains alarmingly high. According to a report from the World Health Organization (WHO) [1], in 2022, NCDs were responsible for 41 million deaths, accounting for 74% of all global deaths, with 40% of these occurring prematurely before the age of 70. This NCD epidemic not only has devastating health consequences for individuals, families, and communities, but also poses a significant burden on healthcare systems worldwide. This burden makes their prevention and control a crucial priority for the 21st century.

Diet plays an important role in the prevention and treatment of NCDs [2]. Unhealthy dietary habits and a lack of knowledge about proper nutrition often contribute to poor diet choices. Fortunately, dietary assessment can help monitor daily food intake and promote healthier eating habits. In recent years, researchers in the field of computer vision and health have shown great interest in dietary assessments [3]. Tools for automating the dietary assessment process have emerged with the widespread use of smartphones with high capacities and the advancements in computer vision models. These tools are known as vision-based dietary assessment (VBDA) systems [4,5,6,7,8]. They utilize image computer vision models to directly identify food items categories, evaluate their volume and estimate nutrient content from smartphone camera pictures. VBDA systems typically involve three stages: food image analysis, portion estimation and nutrient derivation [4]. The performance of the first two stages heavily relies on the effectiveness of artificial intelligence algorithms and the availability of good food datasets, while the final stage depends on a nutritional composition database. The food image analysis stage entails segmenting food regions from the background and recognizing each type of food present in the image. The next step involves evaluating the quantity or volume of each detected food item.

**Figure 1:**
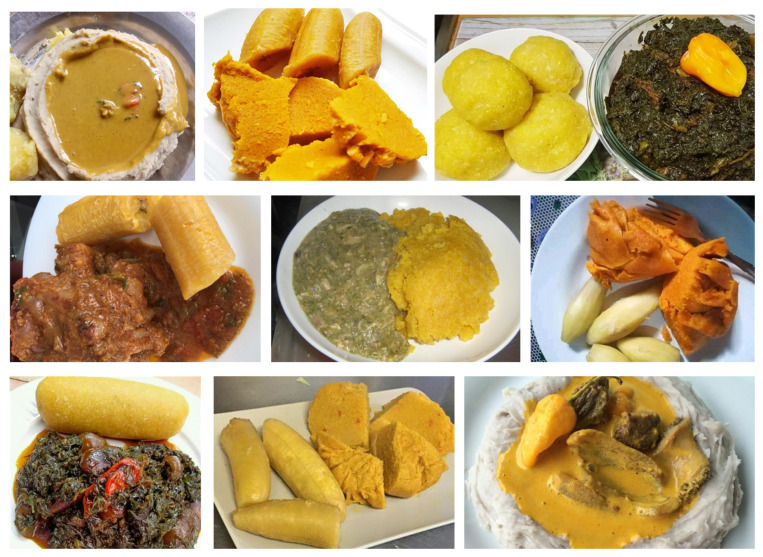
Different kinds of Cameroonian food with a similar yellow texture.

Food image segmentation and recognition indeed pose significant challenges due to various factors. One of the primary challenges is the *non-rigid structure of food*, which differs from common objects. This characteristic makes it difficult to utilize shape as a reliable feature for machine learning models. Additionally, foods usually have high *intra-class variation*, meaning that the visual characteristics of the same food can differ significantly from one to another. This variation is particularly pronounced in African foods, further complicating accurate food recognition. Furthermore, *inter-class resemblance* is another source of potential recognition issues, as different food items can appear very similar, as illustrated in Figure 1. Some examples of generic food with such resemblances include brownies and chocolate cake, and margarine and butter. Moreover, certain dishes may contain various ingredients, resulting in the same dish with distinct visual aspects. Another significant challenge in food image segmentation and recognition is the *scarcity of publicly available datasets* for model training. This lack of datasets hinders the development of accurate segmentation models.

Current research on food image segmentation and recognition focuses mainly on images of Asian and Western foods. Unfortunately, there are only a few publicly available datasets for image segmentation, and none of them incorporate images of African foods, as shown in Table 1. However, African foods, including Cameroonian foods, present their own unique challenges. African dishes often consist of multiple mixed classes of food, as depicted in Figure 1. This complexity adds significant difficulty when attempting to segment and recognize individual food items. The more food classes mixed together on a plate, the more challenging it is to accurately detect the contours of each food component in the dish. In this context, our work specifically focuses on African food, with a particular emphasis on Cameroonian cuisine. The main contributions of our work are described below:

- We propose a novel segmentation model called **mid-DeepLabv3+**. Our model is inspired by the well-known semantic segmentation architecture DeepLabv3+ [9], with three key modifications. Firstly, our backbone is a reduced version of ResNet50, in which we have excluded the last convolution block. This modification reduces the number of parameters of the model but also affects the performance. To recover the loss in performance, we secondly introduce an additional middle layer in the decoder path and thirdly a SimAM [10] attention mechanism. The new middle layer reintroduces more general extracted features that may have potentially been lost in the encoder’s path. These enhancements aim to improve the model’s segmentation performance while making it twice as light as the DeepLabv3+ model with the ResNet50 backbone. The mid-DeepLabv3+ code is shared on Github (https://github.com/babanthierry94/mid-DeepLabv3plus (accessed on 26 December 2023)).
- Public food segmentation datasets are rare, and the construction of a new dataset remains a tedious task, but one that enables us to advance research in the field. We present the first dataset for food image segmentation focusing on African cuisine. This dataset, named **CamerFood10**, includes images of the ten most consumed Cameroonian food classes. It is composed of a total of 1422 images, divided into a training set of 1032 images and a validation set of 209 images. The CamerFood10 dataset is publicly available (https://drive.google.com/drive/u/1/folders/1MugfmVehtIjjyqtphs-4u0GksuHy3Vjz (accessed on 26 December 2023)).

The remainder of this paper is organized as follows: Section 2 describes related work on food image segmentation datasets and techniques. Section 3 presents our dataset CamerFood10 and explains our proposed model architecture, while Section 4 describes the experimental results. The conclusion and future prospects are outlined in Section 5.

## 2. Related Work

### 2.1. Food Image Dataset

With advancements in deep learning models for computer vision, the field of food segmentation and recognition techniques is rapidly evolving. However, the performance of these techniques heavily relies on the availability of large and diverse well-annotated image datasets. Collecting such datasets is a labor-intensive task, and the quality of annotations directly affects the performance of the models.

While some publicly available food image datasets exist, only a few of them are annotated for image segmentation and detection tasks [11,12]. The annotation process for segmentation is particularly tedious and sensitive. Image segmentation datasets vary in terms of geographic origin of the food and the methods used for image collection. Some datasets are annotated with only bounding boxes (UECFOOD100 [13], UECFOOD256 [14]), while others include polygon or mask annotations (MyFood Dataset [15], FoodSeg103 [16], UECFoodPixComplete [17]).

There are four main methods for collecting images for food image datasets. First, images can be captured in a standardized laboratory environment (e.g., UNIMIB2016 [18]), which ensures high-resolution and good-quality images. However, this method typically limits the number of collected images. Second, images can be downloaded from the internet, either from social networks [19] or search engines [15]. This approach allows for the collection of large numbers of images, but can also result in a large number of non-food images that need to be sorted. Downloaded images may vary in quality, including blurry images, images with text, low-resolution images, or retouched images. Third, images can be collected directly from users [20], which provides a realistic representation of real-life scenarios. However, implementing this method can be challenging, as it requires a large number of users and an extended period to collect a substantial amount of images. Finally, some datasets are built with images from other existing datasets. For instance, the UECFoodPixComplete [17] dataset was built by annotating UECFOOD100 [13] images. Likewise, Food201-Segmented is made from Food-101 [21]-segmented images (see Table 1).

Table 1 lists, at the present stage of our investigation, the only publicly available food image datasets for detection and segmentation tasks. These datasets are classified based on their main characteristics, such as their *usage*, *number of classes*, total *number of images*, *method of image collection*, and the *origin* of the dishes represented in the images. Notably, the available datasets mostly focus on Asian or Western foods, and there is currently no dataset available for African foods. As part of our work, we propose the first dataset CamerFood10 specifically designed for image segmentation of African food.

**Table 1:** Summary of publicly available food image datasets for segmentation.

Year	Name	Usage	No. of Classes	No. of Images	Collection Method	Origin
2012	UECFOOD100 [13]	D	100	9060	Web	As, Eu
2014	UECFOOD256 [14]	D	256	31,397	Web	As, Eu
2015	Food201-Segmented [22]	S	201	12,525	Food-101 [21]	USA
2017	School Lunch Dataset [23]	D	21	3940	Normalized	As
2017	UNIMIB2016 [18]	S	73	1027	Normalized	Italia
2019	SUEC Food [24]	S	256	31,995	UECFOOD256	As, Eu
2020	MyFood [15]	S	9	1250	Web	Brazil
2020	Food50Seg [25]	S	50	5000	Web	China
2021	MyFoodRepo-273 [26]	S	273	24,119	Users	Eu
2021	FoodSeg103 [16]	S	103	9490	Web	As, Eu
2021	UECFoodPixComplete [17]	S	102	10,000	UECFOOD100	As, Eu
2022	ChineseDiabetesFood187 [20]	S	187	4052	Users	China
**2023**	**CamerFood10 (ours)**	**S**	**10**	**1241**	**Web**	**Cameroon**

**Usage**: S → segmentation D → detection; **Origin**: As → Asia, Eu → Europe.

### 2.2. Segmentation Model for Food Image

Food image segmentation occurs in the first stage of a VBDA system. Its purpose is to separate food items from the background and from each other. Food image segmentation is a challenging task when food items overlap or do not have strong visual features in contrast with the other food items on a plate. Several methods have been proposed to address issues in food image segmentation. They can be classified in three categories [11]: (i) semi-automatic approaches, (ii) automatic approaches involving machine learning (ML) with handcrafted feature extraction, and (iii) automatic approaches with deep learning feature extraction.

In semi-automatic techniques for food segmentation, the user is asked to select regions of interest in the image or mark some pixels as food items or background. A drawback of the semi-automatic method is that it is tedious. It adds many additional actions for the user, unlike automatic segmentation approaches, where the user only needs to capture the food image. Automatic food image segmentation methods with handcrafted feature (e.g., colour, texture, and shape) extraction rely on traditional image processing techniques [11,27,28], such as region growing and merging [29], Normalized Cuts [30], Simple Linear Iterative Clustering (SLIC), the Deformable Part Model (DPM), the JSEG segmentation algorithm, K-means [31], and GrabCut [32]. One of the most popular works using these techniques was presented by Matsuda and Yanai [13] and takes into consideration the problem of images with multiple foods. It detects several candidate food regions by fusing outputs of several region detectors (DPM, circle detector, and JSEG). Then, it recognizes each candidate region independently using various feature descriptors (SIFT bag, HoG, Gabor textures) and support vector machine (SVM).

With the introduction of deep learning, deep neural networks automatically extract food image features and perform better than methods using traditional image processing techniques [7,11]. Im2Calories [22] was one of the pioneering works that used deep convolutional neural networks (CNNs) for semantic segmentation of food images. Pouladzadeh et al. [33] combined graph-cut segmentation with CNNs for calorie measurement, although their approach was limited to images with a single food label. Some studies have focused on simultaneous localization and recognition of foods using object detection models, like [34], which uses Fast-RCNN, and [35], which uses YOLO. Chiang et al. [36] proposed a model based on a mask region-based convolutional neural network (Mask-RCNN) with a union post-processing technique.

In 2021, Wu et al. [16] proposed a semantic segmentation method consisting of recipe learning (ReLeM) and image segmentation modules. They used a long short-term memory (LSTM) network as an encoder and a vision transformer architecture as a decoder, and they achieved 43.9% mIoU with their dataset FoodSeg103. Okamoto and Yanai [17] used the DeepLabv3+ model on their UECFoodPixComplete dataset and obtained a 55.5% mIoU. Liang et al. [20] introduced a model called ChineseFoodSeg to address challenges specific to Chinese food images, such as blurred outlines, rich colors, and varied appearances. Their model outperformed DeepLabv3+, U-Net, and Mask-RCNN on the ChinesseDiabetesFood187 dataset, achieving an accuracy of 94% and an mIoU of 79%. However, their proposed method is more complex and less time-efficient compared to DeepLabV3+. Sharma et al. [37] proposed a novel architecture named GourmetNet, which incorporates both channel and spatial attention information in expanded multi-scale feature representation using an advanced Waterfall Atrous Spatial Pooling (WASPv2) [38] module with channel and spatial attention mechanisms. GourmetNet achieved state-of-the-art performance on the UNIMIB2016 and UECFoodPix datasets, achieving an mIoU of 71.79% and 65.13% on these datasets, respectively. A more recent work, [39], proposed a Bayesian version of DeepLabv3+ and GourmetNet [37] to perform multi-class segmentation of foods.

It is worth noting that the quality of the image dataset plays a significant role in the performance of these models. Well-arranged food on plates with good clarity often leads to better results. Unfortunately, this is not very often the case in real-world images.

## 3. Proposed Method

### 3.1. CamerFood10 Dataset

The process of building our dataset began by compiling a list of the 42 most consumed foods in Cameroon [40]. To gather a large number of images, a Python script was developed to automatically scrape and download images from search engines (such as Google Image and Bing). We also manually downloaded images from some social media platforms (including Instagram, Twitter, and Facebook). This initial collection resulted in approximately 50,000 images. However, the collected images contained a lot of noise, duplications, and irrelevant classes and were blurry, low resolution, and small-sized.

The next step was to clean up the data. For this purpose, a number of tasks were carried out. Firstly, images with a height or width of less than 400 pixels were removed as they typically lacked sufficient useful information. Duplicate and visually similar images were also eliminated to avoid redundancies. Additionally, non-food-related images were filtered out, focusing solely on food-related content. Blurred and low-quality images were also removed. After that, we ended up with 3067 images. The VGG Image Annotator (VIA) tool [41] was then used to annotate the images with polygon annotations. The CamerFood10 dataset was obtained after further refinement by removing images of classes with very few occurrences. The final dataset consisted of 1241 images and 1513 annotated food items. Our dataset is divided into a training set of 1032 images and a validation set with 209 images.

**Figure 2:**
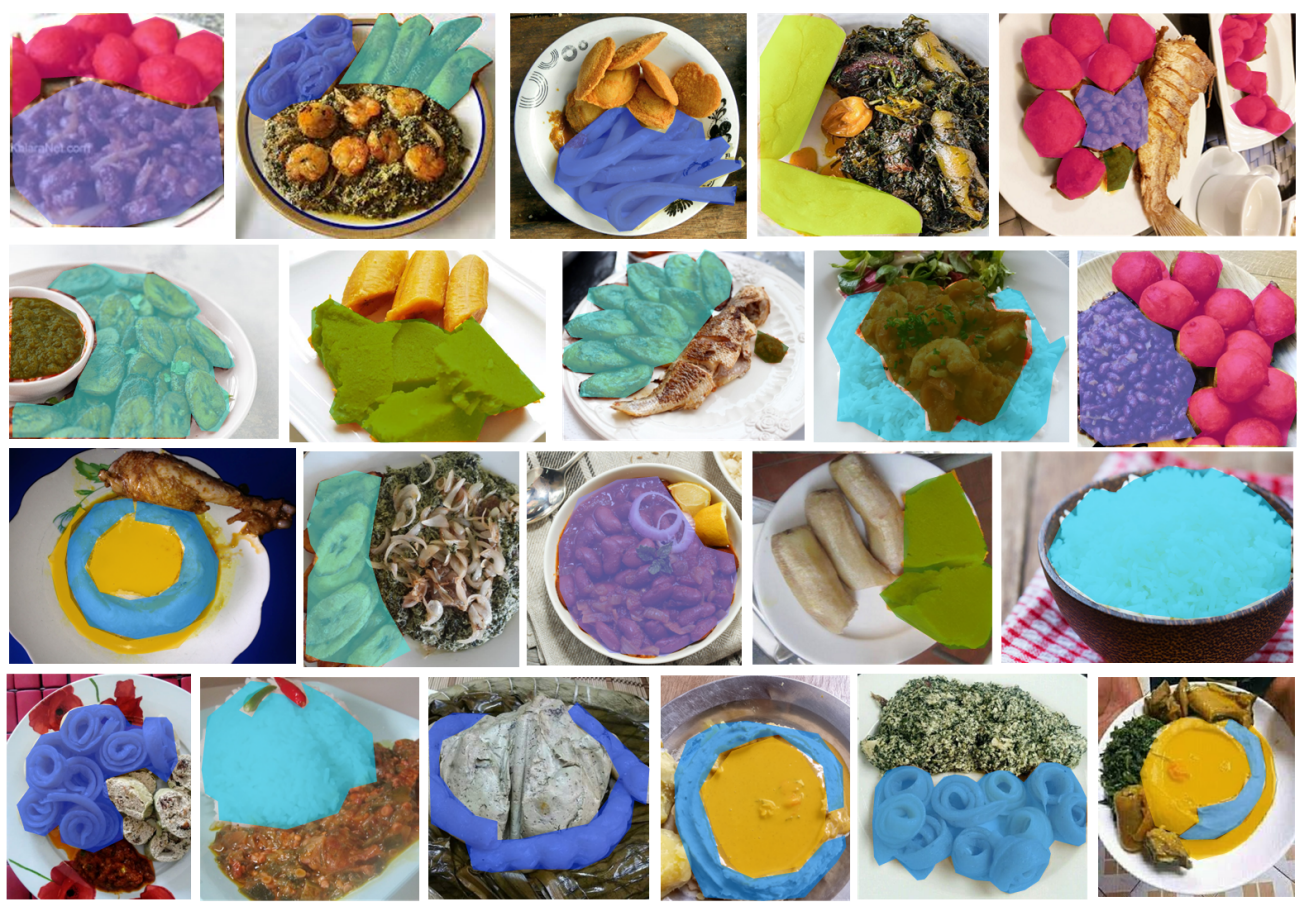
Some images in the CamerFood10 dataset with mask overlay.

**Figure 3:**
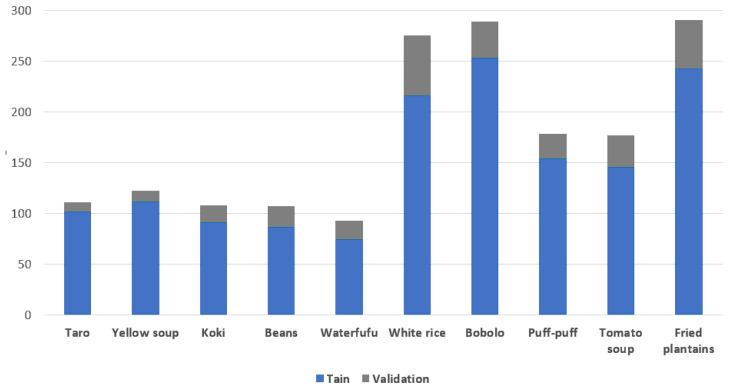
**CamerFood10** class occurrence distribution.

**Figure 4:**
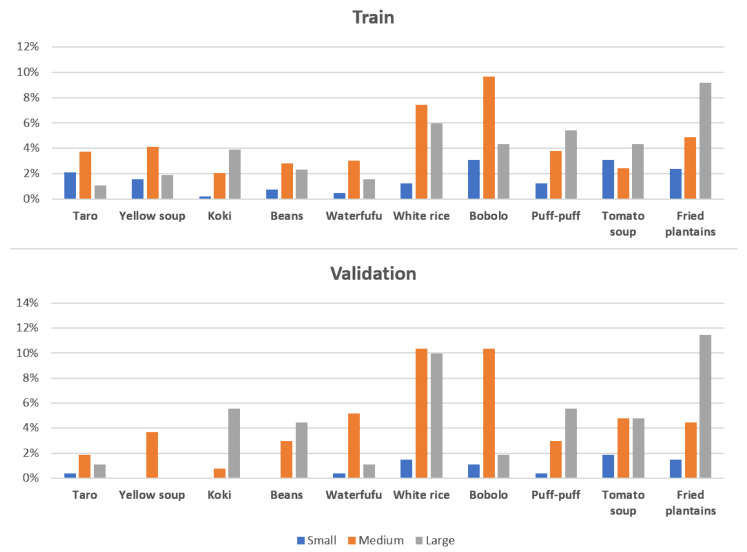
CamerFood10 size distribution of masks from each class based on the number of pixels they occupy in the whole image (i.e., small, medium, large). Small object size < 5% of image; medium object between 5% and 20% of image; large objects > 20% of image.

We present in Figure 2 some examples of CamerFood10 dataset images. We have added masks overlay to the images, illustrating that some CamerFood10 images contain objects from one or more class. We also carried out an exploratory analysis of our dataset, producing the distribution of relative object size, statistics on the number of classes per image, and the number of occurrences per class. Figure 3 illustrates the number of occurrences in each class of the CamerFood10 dataset in the training and validation sets. Our dataset is mainly composed of medium- and large-sized objects distributed as follows: training set (16.09% small objects, 43.95% medium objects, and 39.96% large objects) and validation set (7.01% small objects, 47.23% medium objects, and 45.76% large objects). We define small objects as those whose relative size is less than 5% (length × height) of the entire image, medium objects as those whose size is between 5% and 20% of the image, and large objects as those whose size is greater than 20% of the image. Figure 4 shows the distribution of the CamerFood10 dataset’s relative objects size by class, while Table 2 reports the percentage of the number of classes per image.

The CamerFood10 dataset will serve as a valuable resource for training and evaluating food segmentation models, with a particular focus on African food, specifically Cameroonian cuisine. As shown in Table 1, CamerFood10 is the first publicly available dataset for image segmentation for African food images.

### 3.2. Proposed Model Architecture

**Figure 5:**
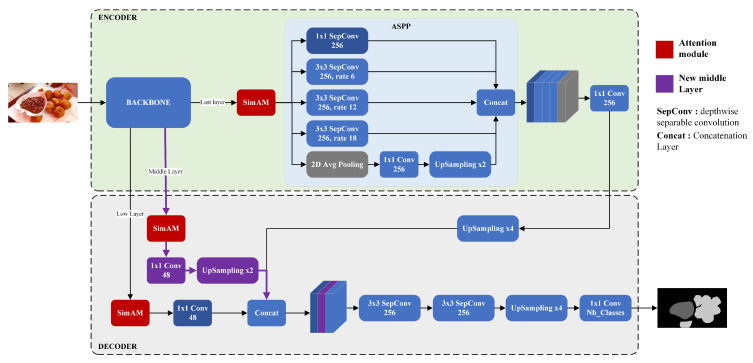
Architecture of our proposed model: mid-Deeplabv3+.

**Figure 6:**
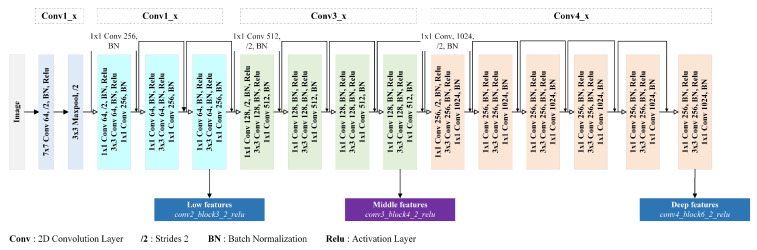
**mid-DeepLabv3+’s** feature extraction backbone based on a scaled-down version of the ResNet50 architecture. This is the ResNet50 model without its fifth convolution block (Conv5).

The architecture of our proposed model is based on DeepLabv3+ [9] with a ResNet50 [42,43] backbone, as depicted in Figure 5 and Figure 6. DeepLabv3+ [9] is a semantic segmentation model developed by Google. It is an encoder–decoder-based network for semantic segmentation with an aligned Xception backbone network for efficient feature extraction.

#### 3.2.1. Backbone and Feature Extraction

Inspired by [44] and its work on road boundary estimation, we employed a reduced ResNet50 [42] network (without the fourth block) for feature extraction. We found that using a backbone model with a small depth yields better results with our dataset. We present in Figure 6 the backbone architecture with the different extracted feature layers. In our proposed mid-DeepLabv3+ model architecture, the shapes of the low, middle, and deep layers are (128×128×64), (64×64×128), and (32×32×256), respectively. In the ResNet50 [42] model, these layers correspond to conv2_block3_2_relu, conv3_block4_2_relu, and conv4_block6_2_relu. By incorporating the middle layer, we effectively introduce additional levels of detail and context to improve the segmentation performance of our model. We denote outputstride as the ratio of input image spatial resolution to the final output resolution (last feature layer). For image semantic segmentation tasks, the outputstride can be equal to 16 or 8. In this work, we adopt an outputstride of 16, as [9] shows that it is the best trade-off between speed and accuracy.

#### 3.2.2. Encoder

The encoder path is the same as DeepLabv3+ [9]. The depth-level feature layer (conv4_block6_2_relu) extracted from the backbone is passed trough an Atrous Spatial Pyramid Pooling (ASPP) [45] module. ASPP captures multi-scale information by incorporating atrous convolutions at different rates. It includes one 1 × 1 convolution, three convolutions with a 3 × 3 kernel size (with dilation rates of 6, 12, 18, respectively), and image-level features with image average pooling. As shown in Figure 5, the resulting features from the five branches are concatenated together and passed through a 1 × 1 convolution layer. Similar to DeepLabv3+ [9], we used *Atrous Separable Convolution* instead of traditional 3 × 3 convolutions in the encoder and decoder paths. This technique factorizes a standard convolution into a depthwise convolution followed by a pointwise convolution (i.e., 1 × 1 convolution), significantly reducing the computational complexity while preserving performance. Then, *Atrous Separable Convolution* (ref. SepConv in Figure 5) is a depthwise 3 × 3 convolution followed by a 1 × 1 pointwise convolution, extra batch normalization, and an ReLU activation layer.

#### 3.2.3. Decoder

In the decoder component of DeepLabv3+ [9], there is a simple but effective approach to refine the segmentation mask by retrieving object segmentation details. First, the low-level features extracted from the encoder backbone (see low layer in Figure 5) undergo 1 × 1 convolution to reduce the number of channels. These reduced features are then concatenated with the encoder output, which is bilinearly upsampled by a factor of 4. The concatenated features are further refined using a 3 × 3 separable convolution layer. Following this, the features are bilinearly upsampled again by a factor of 4. Finally, 1 × 1 convolutions, where the number of filters matches the number of classes, are applied to generate the segmentation result.

One of the challenges with encoder–decoder-based segmentation models is the loss of detailed features during the downsampling process in the encoder and upsampling in the decoder. To address this, our proposed model architecture incorporates an additional extracted middle layer in the encoder backbone, as illustrated in Figure 5. By extracting an intermediate layer, we introduce into the decoder some general characteristics which might be lost in the encoder path and enhance the reconstruction of the semantic mask. The middle layer is processed through 1 × 1 convolution and then bilinearly upsampled by a factor of 2 to match the size of the other reduced feature layers of the decoder. Next, it is concatenated with the features from other branches of the model.

#### 3.2.4. Attention Module

In our mid-DeepLabv3+ proposed model, we further add an attention mechanism after each feature extraction layer. Attention mechanisms have proven to be effective in computer vision, allowing models to focus on relevant parts of the input by assigning different weights to different regions [46]. Several works have demonstrated that incorporating attention can enhance the performance of semantic segmentation models, including DeepLabv3+ [47,48,49]. In our work, we have chosen to use the SimAM (Simple Attention Module) [10]. SimAM, whose Algorithm 1 is shown below, is a lightweight attention module that does not introduce additional parameters. It directly estimates 3D weights, instead of expanding 1D or 2D weights as in other spatial and channel attention mechanisms.

Despite its simplicity, SimAM performs comparably to popular attention mechanisms, while containing no additional parameters. In this work, we tested multiple attention mechanism such as SE (Squeeze-and-Excitation) [50], CBAM [51], BAM [52], ECA [53], Triplet Attention [54], and Coordinate Attention [55]. In our model architecture shown in Figure 5, SimAM is represented by red-colored blocks. This attention module is added after each extracted backbone layer. It enables the model to focus on important features and improve segmentation performance.
**Algorithm 1** Tensorflow-like implementation of SimAM**class SimAM(tf.keras.layers.Layer):**     def __init__(self, lambda = $1\times 10^{-7}$, **kwargs):        super(SimAM, self).__init__(**kwargs)        **self.lambda = lambda**     def call(self, X, **kwargs):        *# X Input feature [N, H, W, C]*        **height, width = X.shape[1:2]**        *# spatial size*        n = width * height − 1        *# square of (X − u)*        **u = tf.math.reduce_mean (X, axis = (1,2), True)**        **d = tf.math.square (X − u)**        *# d.sum()/n is channel variance*        **v = tf.math.reduce_sum (d, axis = (1,2), True)/n**        *# E_inv groups all importance of X*        E_inv = d/(4 * tf.maximum (v, self.lambda) + 0.5)        *# return attended features*        return X * tf.keras.activations.sigmoid(E_inv)

## 4. Experiments and Results

To evaluate our work, we first compare the performance of our proposed model on our dataset with other popular CNN benchmark models for semantic image segmentation. Additionally, we tested our model on the publicly available MyFood dataset [15] and compared the results with their best reported results. The evaluation metrics used include mean Intersection Over Union (mIOU) and mean Pixel Accuracy (mPA). Furthermore, we computed the mIoU for each class in the CamerFood10 dataset to assess its balance.

### 4.1. Compared Approaches

We compared the performance of the mid-DeepLabv3+ model with some popular CNN semantic image segmentation models, including GourmetNet [37], DeepLabv3+ [9], FCN [56], ResUnet [57], and U-Net [58].

#### 4.1.1. U-Net

Ref. [58] details a commonly used architecture for semantic image segmentation. It has two paths: a contracting (encoder) path and an expansive (decoder) path. The contracting path follows the typical architecture of a convolutional network. Each stage of the expansive path consists of an oversampling of the feature map followed by 2 × 2 up-convolution, which halves the number of feature channels; a concatenation with the corresponding cropped feature map from the contracting path; and two 3 × 3 convolutions, each followed by ReLU. Finally, 1 × 1 convolution is applied to match each 64-component feature vector to the desired number of classes. U-Net has demonstrated impressive performances in a wide range of image segmentation tasks, including medical image analysis and satellite imagery, among others. In our work, we used VGG16 [59] as the encoder path.

#### 4.1.2. GoumetNet

Ref. [37] details a novel architecture and was published by Sharma et al. in 2021. It incorporates both channel attention and spatial attention information in an expanded multi-scale feature representation using an advanced Waterfall Atrous Spatial Pooling (WASPv2) module [38]. GourmetNet refines the feature extraction process by merging features from multiple levels of the backbone through two attention modules. The refined features are processed with the advanced multi-scale waterfall module that combines the benefits of cascade filtering and pyramid representations.

#### 4.1.3. FCN-8

Ref. [56] details a module that can be added to a feature extractor network. The primary idea behind FCN-8 is to convert classification networks (like VGG, ResNet, etc.) into a fully convolutional architecture to achieve dense predictions at the pixel level. In our experiments, we used FCN-8 with VGG-16 and ResNet50 as a backbone network.

#### 4.1.4. ResUnet

Ref. [57] details the deep Residual U-Net. It is an encoder–decoder architecture for semantic segmentation, initially used for road extraction from high-resolution aerial images in the field of remote sensing image analysis. It combines the strengths of residual learning and U-Net. The network combines the deep residual units and skip connections of the U-Net architecture. The multiple skip connections within the network can facilitate information propagation. It is an improvement over the existing U-Net architecture with fewer parameters.

#### 4.1.5. DeepLabv3+

Ref. [9] details a semantic segmentation model designed by the Google research group in 2016. The model employs Aligned Xception in the encoder path to extract high-level features from the input image. These features are then processed through an Atrous Spatial Pyramid Pooling (ASPP) module, which captures multi-scale contextual image information. This helps the model understand the global context of the image and improves its ability to handle objects at different scales. In the decoder part, DeepLabv3+ uses a combination of bilinear upsampling and skip connections to recover the spatial resolution of the feature map. DeepLabv3+ replaces 3 × 3 convolution by depthwise separable convolution with Atrous Spatial Pyramid Pooling, the Xception backbone, and decoder modules. In this work, we compared our model to DeepLabv3+ with three different backbones: Xception, ResNet50, and ResNet101.

### 4.2. Evaluation Metrics

Semantic segmentation models aim to predict the class of each pixel in an image. Several metrics have been discussed in the literature and their usage depends on the specific requirements of the application. A model may perform well under one metric but poorly under another one. As presented in [11], the main metrics for segmentation model evaluation are mean Intersection over Union (mIoU) and Pixel Accuracy (mPA). These metrics are normalized and range between 0 and 1, where a value of 0 indicates poor performance. Note that True Positive pixels represent pixels which have been correctly predicted to belong to the given class (according to the target mask) whereas a True Negative represents a pixel that has been correctly identified as not belonging to the given class. In Equations (1) and (2) below, *N* is the total number of food classes and $TP_{i}$, $TN_{i}$, $FP_{i}$, $FN_{i}$ are, respectively, the total number of True Positive, True Negative, False Positive, and False Negative pixels for the class *i*.

#### 4.2.1. Mean Pixel Accuracy (mPA)

The mean Pixel Accuracy is the ratio of pixels correctly classified (see Equation (1)). This metric is easy to understand but a high accuracy does not necessary imply superior segmentation ability. This metric does not fit imbalanced datasets:

(1) $$\mathrm{mPA}=\frac{1}{N}\sum_{i=1}^{N}\frac{TP_{i}+TN_{i}}{TP_{i}+TN_{i}+FP_{i}+FN_{i}}.$$

#### 4.2.2. Mean Intersection Over Union (mIoU)

The mean Intersection Over Union is one of the most commonly used metrics for multi-class semantic segmentation. It measures the average overlap between the predicted segmentation masks and the ground truth masks across all classes:

(2) $$\mathrm{mIoU}=\frac{1}{N}\sum_{i=1}^{N}\frac{TP_{i}}{TP_{i}+FP_{i}+FN_{i}}.$$

### 4.3. Experimental Environment

We conducted our experiments using a compute node on Grid5000 [60] equipped with a GeForce GTX 1080 Ti (NVIDIA Corporation, Santa Clara, CA, USA). Tensorflow (v. 2.14.0-rc1), Keras API (3.0.1), and Python 3 were utilized for all experiments. During the training phase, we employed an input image size of 512 px × 512 px, the standard *Adam* optimizer, and *categorical cross-entropy* loss. To adjust the learning rate, we adopted the *ReduceOnPlateau* learning schedule with an initial learning rate of 1 × $10^{-4}$. The validation loss was monitored, and we set the patience parameter to 25 and the reduce factor to 0.8. The models were trained for 250 epochs on the CamerFood10 dataset. All datasets and the code of each model used in this paper are publicly available here (https://github.com/babanthierry94/mid-DeepLabv3plus (accessed on 26 December 2023)).

### 4.4. Results

In this section, we present the results obtained for different experiments we performed to validate our contribution.

#### 4.4.1. mid-DeepLabv3+ Performance Analysis

In this experiment, we explore how different contributions impact the model performances. We first evaluated the performance metrics for DeepLabV3+ with the reduced ResNet50 (see Figure 6) backbone, which we consider our baseline model. Then, we tested different version of the proposed model by alternately adding the SimAM at different places in the baseline network: the middle layer (*Mid-Layer*) and both at the same time. Our proposed model, named mid-DeepLabv3+, was obtained by adding both SimAM after the backbone-extracted layers (see Figure 3). In Table 3, we illustrate the improvement achieved by each contribution over the baseline model, DeepLabv3+ with a ResNet50 backbone. This table provides a comparison based on the evaluation metrics *mIoU* and *mPA*. We observe that the inclusion of the middle layer plays the main role in improving the model’s performance. It enables the model to capture additional contextual information and refine the segmentation results. On the other hand, the attention mechanism contributes to reasonably improving the model with our proposed middle layer.

#### 4.4.2. Performance with Different Attention Mechanisms

In this experiment, we tested some benchmark attention mechanisms on our proposed mid-DeepLabv3+ model. We tested multiple attention mechanisms, such as SimAM [10], SE (Squeeze-and-Excitation) [50], CBAM [51], BAM [52], ECA [53], Triplet Attention [54], and Coordinate Attention [55]. Table 4 presents the results obtained with our model using different attention mechanisms. As shown in Table 4, SimAM has a better performance over other benchmark attention mechanisms, while having the lowest number of parameters.

#### 4.4.3. Comparison with Other CNN Benchmark Models

**Figure 7:**
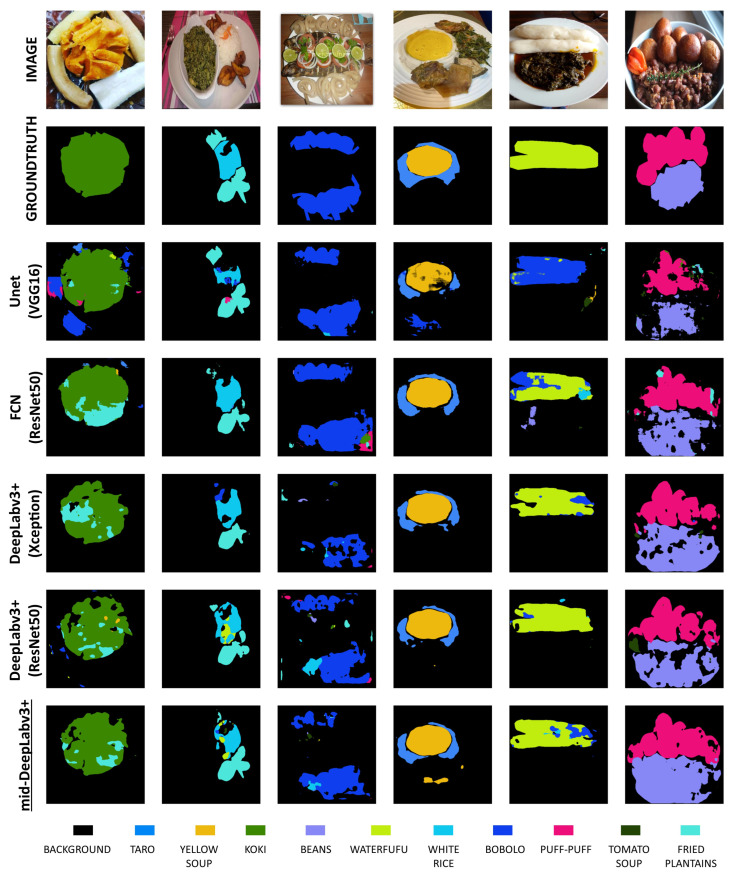
Several images in the CamerFood10 dataset and ground truth mask and prediction with mid-DeepLabv3+ and other benchmark models. For spatial reasons, we only present the predictions of the models with the best results.

This third experiment was conducted to assess how our model performs in comparison to other popular benchmark models for image segmentation on the same dataset. We compared our model to DeepLabv3+ [9], GourmetNet [37], and Unet [58]. As shown in Table 5, for DeepLabv3+, we tested Aligned Xception [9], as presented in the original paper, and ResNet101 [42] and ResNet50 [42] as presented in [45]. All the models were built with outputstride=16. Table 5 reports the results obtained using the CamerFood10 dataset. The mid-DeepLabv3+ model surpasses benchmark models on our dataset. The FCN-8 model with a ResNet50 backbone presents promising results but remains too heavy with a higher number of parameters and FLOPS (floating point operations per second). Despite the good results presented by GourmetNet [37], in their related paper, this model presented very poor results on our dataset. The lower performance of DeepLabv3+ with ResNet101 and Xception backbones compared to DeepLabv3+ with a ResNet50 backbone can be attributed to the complexity and depth of these models. It should be noted that models with more layers and parameters tend to require a huge number of images for training. Therefore, our main comparison focused on results obtained with the same ResNet50 [42] backbone. Our proposed model, mid-DeepLabv3+, achieved a higher accuracy while containing less parameters relative to the other benchmark models we explored. Compared to the standard DeepLabv3+ with a ResNet50 [42] backbone, our model has twofold fewer learning parameters, while a +10.74% improvement in the mIoU on the CamerFood10 dataset. On the other hand, our model has a higher number of FLOPS than the standard DeepLabv3+ model. Figure 7 shows predictions of the different tested models.

#### 4.4.4. Evaluation on Another Dataset

In this experiment, the performance of the proposed model was evaluated when tested on other datasets. For this purpose, we chose the MyFood dataset published by Freitas et al. [15], made up of images of the most consumed Brazilian food. The MyFood dataset has nine classes and contains 1250 images (see Table 1). With MyFood, we trained the same models as with CamerFood10 in the previous experiment. The obtained results are reported in Table 6. We found that our results were comparable to those obtained by Freitas et al. [15]. We observed that our model, mid-DeepLabv3+, outperformed benchmark models on the MyFood dataset.

#### 4.4.5. CamerFood10 Class Performance Analysis

Finally, we computed the mIoU for each class of our CamerFood10 dataset in order to further investigate how the proposed model performs for each class of food. In Table 7, we provide the mIoU results for each class of CamerFood10. The classes are ranked from the highest mIoU to the lowest. Upon analyzing the results, we observed a low performance for classes with very few occurrences (as shown in Figure 3), such as “*Taro*”, “*Yellow soup*”, “*Koki*”, and “*Beans*”. We also obtained lower results for classes with high intra-class variation, such as “*Tomato soup*” and “*Bobolo*”. On the other hand, higher results were achieved for classes with many occurrences and minimal intra-class variation, such as “*White rice*”, “*Puff-puff*”, and “*Fried plantain*”. Figure 7 shows some predicted masks obtained with mid-DeepLabv3+.

## 5. Conclusions and Discussion

This paper addresses the issue of developing a vision-based dietary assessment (VBDA) system for African foods. Focusing on the image analysis phase, we have made two main contributions. We built a food image segmentation dataset CamerFood10, which is the first publicly available dataset for African food. With publicly available datasets on food segmentation being scarce (as shown in Table 1), we are convinced that this dataset will be a valuable asset for researchers in the field of automatic dietary assessment, as African foods have their own specificity, as we have shown earlier in this paper. Next, we proposed a new model, mid-DeepLabv3+, for image segmentation. This model is based on DeepLabv3+ and uses SimAM after backbone feature extraction combined with a new middle layer in the decoder path. Mid-DeepLabv3+ also uses a reduced version of ResNet50 as its backbone, allowing it to have fewer parameters and be lighter. Our model outperformed some popular CNN benchmark models for image segmentation, achieving an mIoU value of 65.20% on CamerFood10. Compared to DeepLabv3+ with the same backbone, mid-DeepLabv3+ achieved a +10.74% mIoU performance improvement while having twofold fewer parameters. However, while CNN segmentation models such as our mid-DeepLabv3+ model perform well, they still have some limitations. CNNs may require a large amount of labeled data for training, especially in tasks like food image segmentation where learning complex hierarchical features is necessary. Furthermore, CNNs operate on local receptive fields and may not capture the global context effectively. This limitation can impact tasks where understanding the entire context is crucial. This may be the case where using global context can help to tackle one of the major challenges of food segmentation, namely “*intra-class*” confusion. New computer vision techniques such as transformers use self-attention mechanisms to capture long-range dependencies and capture the overall context more effectively. As part of our future work, we intend to, on the one hand, expand the number of images in the dataset and, on the other hand, investigate methods using full or hybrid transformer-based models. In addition to accurately estimating the calories of the food in a given image, we also plan to explore methods for estimating the amount of each food item.

## Figures and Tables

**Table 2:** CamerFood10 distribution of the number of classes per image. The background class is not taken into account in the distribution.

	One Class	Two Classes	Three Classes	Four Classes
Training	76.65%	22.09%	1.16%	0.10%
Validation	81.82%	17.70%	0.48%	0.0%

**Table 3:** Results of mid-DeepLabv3+ ablation experiments for various configurations on the CamerFood10 dataset.

Architecture	mIoU (%)	mPA (%)
DeepLabv3+ (ResNet50) [9]	58.88	88.73
Baseline	62.46	89.87
Baseline and SimAM	64.13	90.37
Baseline and Mid-Layer	65.17	90.46
**Baseline and Mid-Layer and SimAM (ours)**	**65.20**	**90.47**

**Table 4:** Results of mid-DeepLabv3+ with benchmark attention mechanisms on the CamerFood10 dataset.

Attention Mechanism	mIoU (%)	mPA (%)	#Params
**Our model + SimAM [10]**	**65.20**	**90.47**	**10,406,762**
ECA [53]	62.60	89.79	+2,100
CoordAtt [55]	62.41	89.83	+900
TripletAtt [54]	62.20	89.88	+11,800
SE [50]	64.33	90.27	+11,200
BAM [52]	63.78	90.03	+33,200
CBAM [51]	60.79	89.11	+11,500

**Table 5:** mid-DeepLabv3+ results and comparison with state-of-the-art models on the CamerFood10 dataset.

Model	Backbone	mIoU (%)	mPA (%)	#Params (M)	#FLOPS (M)
DeepLabv3+ [9]	ResNet50	58.88	88.73	27.91	1397.47
DeepLabv3+ [9]	ResNet101	51.67	86.48	46.98	1785.36
DeepLabv3+ [9]	Xception	58.15	88.27	42.19	**755.45**
GourmetNet [37]	ResNet101	27.52	76.24	47.39	2549.08
Unet [58]	VGG16	49.74	87.86	25.86	4372.14
FCN-8 [56]	ResNet50	61.88	89.67	451.51	2756.86
FCN-8 [56]	VGG16	49.18	85.01	134.35	790.44
ResUnet [57]	–	43.79	85.23	**8.23**	3621.53
**mid-DeepLabv3+**	**ResNet50**	**65.20**	**90.47**	10.41	1465.92

ResUnet is not built with a standard backbone model for feature extraction.

**Table 6:** mid-DeepLabv3+ results and comparison with state-of-the-art models on the MyFood [15] dataset.

Model	Backbone	mIoU (%)	mPA (%)
DeepLabv3+ [9]	ResNet50	68.10	88.81
DeepLabv3+ [9]	ResNet101	65.61	87.74
DeepLabv3+ [9]	Xception	62.24	85.81
GourmetNet [37]	ResNet101	30.12	70.70
Unet [58]	VGG16	56.94	83.07
FCN-8 [56]	ResNet50	63.67	87.15
FCN-8 [56]	VGG16	52.53	80.87
ResUnet [57]	–	43.13	76.57
**mid-Deeplabv3+**	**ResNet50**	**69.23**	**89.26**

ResUnet is not built with a standard backbone model for feature extraction.

**Table 7:** Analysis of mid-Deeplabv3+’s classwise segmentation performance for CamerFood10. Classes are ranked from highest mIoU to the lowest.

Class Name	mIoU (%)
White rice	77.84
Fried plantain	76.12
Puff-puff	72.41
Koki	63.80
Tomato soup	61.88
Waterfufu	60.57
Taro	58.98
Bobolo	53.69
Yellow soup	53.44
Beans	48.95

## Data Availability

The data presented in this study are openly available in this repository https://drive.google.com/drive/u/1/folders/1MugfmVehtIjjyqtphs-4u0GksuHy3Vjz (accessed on 26 December 2023).
